# The Mechanism and Clinical Significance of Circular RNAs in Hepatocellular Carcinoma

**DOI:** 10.3389/fonc.2021.714665

**Published:** 2021-09-02

**Authors:** Ziyue Huang, Haoming Xia, Shuqiang Liu, Xudong Zhao, Risheng He, Zhongrui Wang, Wenguang Shi, Wangming Chen, Pengcheng Kang, Zhilei Su, Yunfu Cui, Judy Wai Ping Yam, Yi Xu

**Affiliations:** ^1^Department of Hepatopancreatobiliary Surgery, Second Affiliated Hospital of Harbin Medical University, Harbin, China; ^2^Department of Pathology, Li Ka Shing Faculty of Medicine, The University of Hong Kong, Hong Kong, Hong Kong, SAR China; ^3^The Key Laboratory of Myocardial Ischemia, Harbin Medical University, Ministry of Education, Harbin, China

**Keywords:** circular RNAs, hepatocellular carcinoma, sponge, biomarker, diagnosis, prognosis

## Abstract

Hepatocellular carcinoma (HCC) is one of the most prevalent malignant tumors worldwide. In view of the lack of early obvious clinical symptoms and related early diagnostic biomarkers with high specificity and sensitivity, most HCC patients are already at the advanced stages at the time of diagnosis, and most of them are accompanied by distant metastasis. Furthermore, the unsatisfactory effect of the follow-up palliative care contributes to the poor overall survival of HCC patients. Therefore, it is urgent to identify effective early diagnosis and prognostic biomarkers and to explore novel therapeutic approaches to improve the prognosis of HCC patients. Circular RNA (CircRNA), a class of plentiful, stable, and highly conserved ncRNA subgroup with the covalent closed loop, is dysregulated in HCC. Increasingly, emerging evidence have confirmed that dysregulated circRNAs can regulate gene expression at the transcriptional or post-transcriptional level, mediating various malignant biological behaviors of HCC cells, including proliferation, invasion, metastasis, immune escape, stemness, and drug resistance, etc.; meanwhile, they are regarded as potential biomarkers for early diagnosis and prognostic evaluation of HCC. This article reviews the research progress of circRNAs in HCC, expounding the potential molecular mechanisms of dysregulated circRNAs in the carcinogenesis and development of HCC, and discusses those application prospects in the diagnosis and prognosis of HCC.

## Introduction

Hepatocellular carcinoma is the sixth most common hepatic malignancy and causes severe burden of mortality, making it rank as the third leading cause of cancer-associated deaths worldwide, with approximately 906,000 new cases and 830,000 deaths (1, 2). Epidemiological and experimental studies have demonstrated that the initiation and progression of HCC may be caused and promoted by hepatitis B virus (HBV) or hepatitis C virus (HCV), aflatoxin-contaminated foods, non-alcoholic fatty liver disease (NAFLD), excessive drinking, genetic factors, smoking, excess body weight, type 2 diabetes (3). At present, there are still many limitations to existing diagnostic methods for HCC. On the one hand, the classical biomarkers for clinical diagnosis lead to some false-positive and false-negative results in HCC diagnosis, such as alpha-fetoprotein (AFP) was widely used in early detection of HCC, but it may also appear in varying degrees in liver diseases such as hepatitis and liver cirrhosis (LC); on the another hand, liver electronic computer tomography (CT) and magnetic resonance imaging (MRI) show unclear nodules <2 cm (4, 5). Currently, the main methods of treatment for HCC are liver resection, liver transplantation, percutaneous thermal ablation, radiotherapy, chemotherapy, and immunotherapy (6). Among them, surgical resection is the first-choice treatment for HCC. Because of the lack of effective biomarkers and insidious early clinical symptoms, most HCC patients are at the advanced stage when diagnosed, losing the opportunity of radical resection; furthermore, other palliative treatment options remain no satisfactory survival benefits, leading to the poor clinical prognosis of HCC patients. Therefore, it is extremely necessary to clarify the molecular mechanisms of the oncogenesis and development of HCC, find accurate and potent biomarkers for early diagnosis and prognostic prediction, and formulate effective HCC treatment strategies.

Approximately 93% of the DNA sequences of the eukaryotic organism genome can be transcribed into RNA, of which only 2% can be translated into protein, while 98% are non-coding RNAs (ncRNAs) with no protein coding capability or very low coding capability. The development of high-throughput RNA sequencing led to the discovery of a large number of ncRNAs. The number of identified ncRNA genes exceeds that of coding transcripts (7–10). According to the length of the transcript, ncRNAs can be divided into short ncRNAs (<200 nucleotides) and long non-coding RNAs (lncRNAs, >200 nucleotides) (11). Researches have shown that the dysregulated lncRNAs and short ncRNAs can play a vital role in regulating malignant tumor-related genes. For example, lncRNAs can interact with DNA, RNAs, or proteins, regulating gene expression at the transcription and post-transcriptional levels (12–14). MicroRNAs (MiRNAs) can specifically bind to target mRNAs, leading to mRNA degradation or inhibiting protein translation (15).

**Figure 1:**
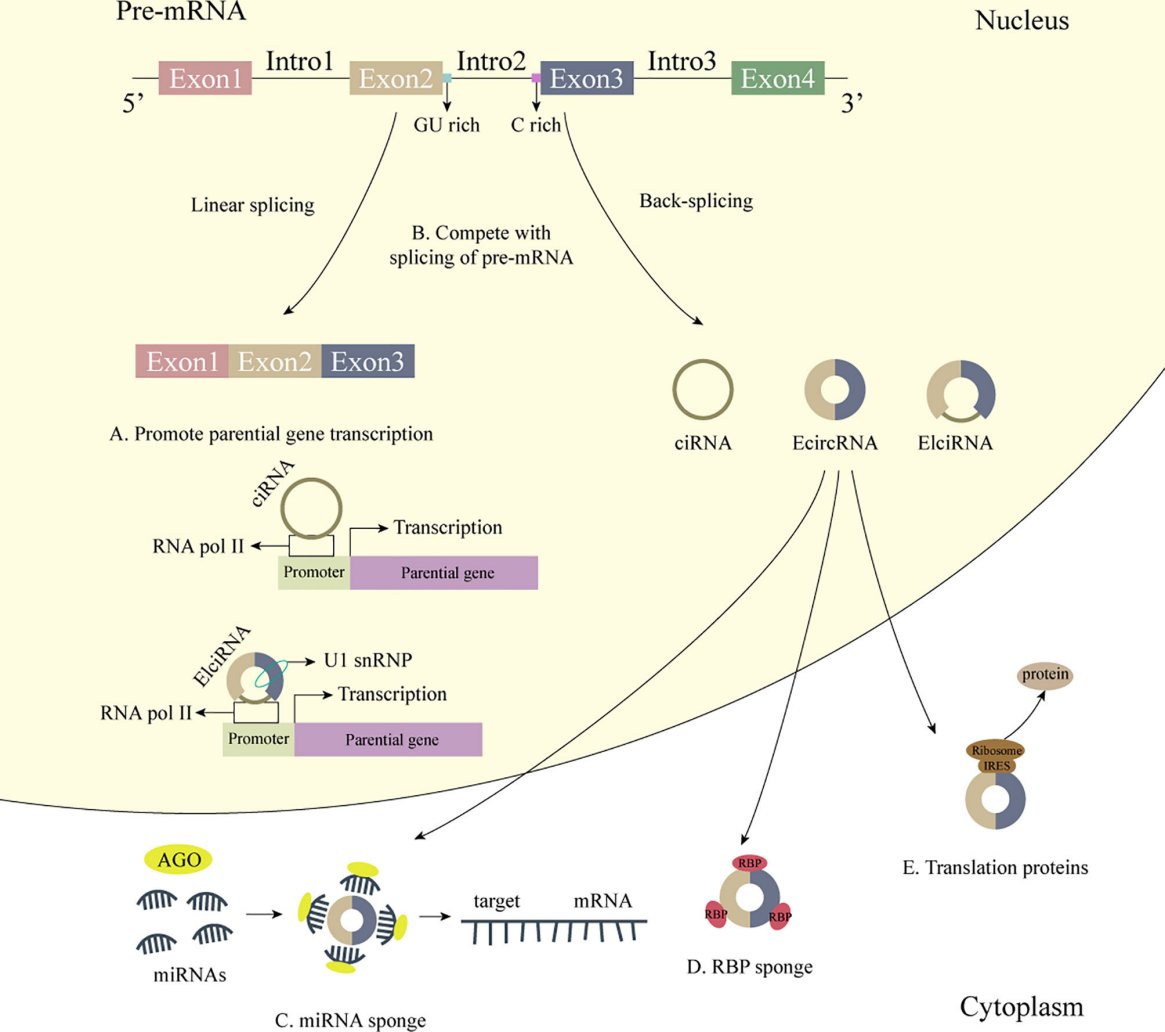
The biogenesis and biological functions of circRNA. 1) The biogenesis of circRNA. CircRNAs are exon or intron sequences spliced in reverse from the pre-mRNA. 2) Some biological functions of circRNA. **(A)** CiRNAs interact with RNA Pol II, and EIciRNAs interact with RNA Pol II and U1 snRNP and further promote the transcription of their parental genes. **(B)** CircRNAs affect the selective splicing of pre-mRNA. **(C)** CircRNAs act as miRNA sponge. **(D)** CircRNAs act as RBP sponge. **(E)** CircRNAs bind to IRES to generate functional proteins.

Over the past few years, circRNA, as a new type of ncRNA, has been widely concerned. Unlike traditional linear RNA, circRNA has a covalently closed, continuous loop structure without a 5’ cap and a 3’ tail, which makes it resistant to ribonuclease cleavage and expresses itself in a stable manner (16–19). Meanwhile, most circRNAs show high tissue specificity and developmental stage-related expression patterns, as well as a high conservation among species (20–22). Based on these characteristics, circRNAs also have great potential in disease diagnosis, progress monitoring, prognosis prediction, etc. CircRNAs are exon or intron sequences spliced in reverse from the precursor mRNA (pre-mRNA), which can be classified as exon circular RNAs (EcircRNAs), intron circular RNAs (ciRNAs), and exon-intron circular RNAs (EIciRNAs) (21). Among them, EcircRNAs account for the majority of circRNAs, which mainly exist in the cytoplasm, acting as miRNA sponge (23, 24), interacting with RNA binding protein (RBP) (25), or encode proteins (26); while ciRNAs and EIciRNAs are widely present in the nucleus, which can act as transcription/translation regulators (27) or affect the selective splicing of pre-mRNA (28) **(** **Figure 1** **)**.

Additionally, emerging evidence have indicated that circRNAs are involved in mediating the tumorigenesis and development of various tumors, exhibiting great potential as a molecular target of cancer therapy. For example, circSEPT9 regulated by E2F transcription factor 1 (E2F1) and eukaryotic translation initiation factor 4A3 (EIF4A3) pushes the cancerous derivation of triple-negative breast cancer through the circSEPT9/miR-637/Leukemia Inhibitory Factor (LIF) axis (29). CircNRIP1 acts as a microRNA-149-5p sponge to promote gastric cancer progression through the AKT1/mTOR pathway (30). CircMRPS35 can specifically bind to the forkhead box protein O1/3a (FOXO1/3a) promoter region to activate its transcription, subsequently triggering the expression of downstream target genes p21, p27, Twist1, and E-cadherin, thereby inhibiting the malignant biological behaviors of tumors (31). In this review, we summarized the functions and mechanisms of circRNAs in the oncogenesis and malignant progression of human HCC.

## Role of CircRNAs in HCC

**Figure 2:**
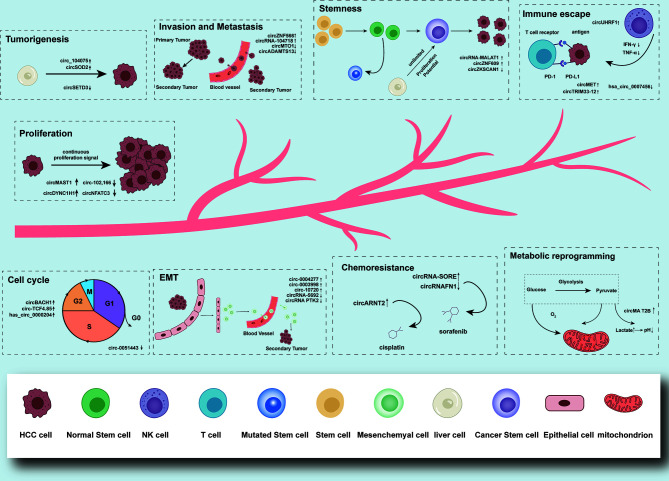
Functions and brief mechanism of circRNAs in HCC tumorigenesis and progression. Aberrantly expressed circRNAs in HCC can participate in the mediation of tumorigenesis, cell proliferation and apoptosis, invasion and metastasis, cell cycle, EMT, immune escape, drug resistance, metabolic reprogramming, and stemness.

It is reported that circRNA, which is unregulated expressed in tumor tissues, plays a role in the promotion or suppression in the development of tumors. CircRNAs can participate in the mediation of tumorigenesis, cell proliferation and apoptosis, invasion and metastasis, cell cycle, epithelial-mesenchymal transition (EMT), immune escape, drug resistance, metabolic reprogramming, and other malignant biological behaviors in HCC patients, and can be used as HCC potential biomarkers for early diagnosis and clinical prognostic prediction **(** **Figure 2** **)**. In the following section, the role of circRNAs in the oncogenesis and development of HCC and the molecular mechanisms involved will be briefly described, and the application prospects of circRNAs in HCC diagnosis and prognostic evaluation will be described **(** **Table 1** **)**.

**Table 1:** Representative circRNAs and related signaling pathways in HCC.

CircRNA	Current circBase ID	Genomic position	Spliced length (bp)	Expression	Biological functions	Regulatory axis	References
circSOD2	hsa_circ_0004662	chr6:160103505-160109274	462	up	promotes tumorigenesis	miR-502-5p/DNMT3a/JAK2/STAT3	(32)
circ_104075	hsa_circ_0075736	chr6:17669523-17669777	162	up	promotes tumorigenesis	miR-582-3p/YAP	(33)
circTMEM45A	hsa_circ_0066659	chr3:100274052-100296285	1254	up	promotes tumorigenesis	miR-665/IGF2	(34)
circ_0001955	hsa_circ_0001955	chr15:64495280-64508912	815	up	promotes tumorigenesis	miR-516a-5p/TRAF6/MAPK11	(35)
hsa_circ_0016788	hsa_circ_0016788	chr1:228581376-228594517	2691	up	promotes tumorigenesis	miR-486/CDK4	(36)
circSETD3	hsa_circ_0000567	chr14:99924615-99932150	683	down	inhibits tumorigenesis	miR-421/MAPK14	(37)
SCD-circRNA 2	/	/	/	up	enhances proliferation	SCD-circRNA 2/ERK	(37)
circBACH1	hsa_circ_0061395	chr21:30698379-30702014	1542	up	promotes proliferation, controls cell cycle	HuR/p27	(39)
hsa_circ_0000204	hsa_circ_0000204	chr10:1117078-1118233	1155	up	promotes proliferation, controls cell cycle	miR-191/KLF6	(40)
circRNA-100,338	/	/	/	up	promotes angiogenesis, migration	circRNA-100,338/NOVA2	(41)
circPTGR1 isoform	hsa_circ_0003731	chr9:114341075-114348445	442	up	promotes invasion, migration	miR-449a/MET	(42)
	hsa_circ_0008043	chr9:114332370-114348445	670				
	hsa_circ_0088030	chr9:114337013-114348445	551				
circASAP1	hsa_circ_0085616	chr8:131370262-131374017	229	up	promotes proliferation, invasion, migration, colony formation, TAM infiltration	miR-326/miR-532-5p/MAPK1/ERK1/2,miR-326/miR-532-5p/CSF-1	(43)
circMAST1	hsa_circ_0049613	chr19:12962747-12963209	303	up	promotes proliferation, invasion	miR-1299/CTNND1	(44)
circDYNC1H1	hsa_circ_0033351	chr14:102499401-102507010	1862	up	promotes proliferation, migration	miR-140-5p/SULT2B1	(45)
circSLC3A2	hsa_circ_0022587	chr11:62650379-62653080	669	up	promotes proliferation, invasion	miR-490-3p/PPM1F	(46)
circHIPK3	hsa_circ_0000284	chr11:33307958-33309057	1099	up	promotes proliferation, migration	miR-124/AQP3	(47)
hsa_circ_0001955	hsa_circ_0001955	chr15:64495280-64508912	815	up	promotes proliferation, invasion, migration	miR-145-5p/NRAS	(48)
circ-TCF4.85	/	/	/	up	promotes proliferation, invasion, migration, cell cycle, inhibits apoptosis	miR-486-5p/ABCF2	(49)
circZNF566	hsa_circ_0141434	chr19:36940300-36940903	603	up	promotes proliferation, invasion, migration	miR-4738-3p/TDO2	(50)
hsa_circRNA 103809	hsa_circ_0072088	chr5:32379220-32388780	693	up	promotes proliferation, migration, cell cycle	miR‐377‐3p/FGFR1	(51)
hsa_circ_104348	/	/	/	up	promotes proliferation, invasion, migration, inhibits apoptosis	miR-187-3p/RTKN2/Wnt/β-catenin	(52)
circRNA-104718	/	/	/	up	promotes growth, migration	miR-218-5p/TXNDC5	(53)
circFBLIM1	hsa_circ_0010090	chr1:16084668-16113084	3935	up	promotes growth, invasion, inhibits apoptosis	miR-346/FBLIM1	(54)
circ-DB	hsa_circ_0025129	chr12:6450941-6451283	342	up	promotes growth, invasion, modulates DNA damage	miR-34a/USP7/Cyclin2	(55)
circRASGRF2	hsa_circ_0073181	chr5:80338695-80409739	2182	up	promotes proliferation, invasion, migration, cell cycle, inhibits apoptosis	miR-1224/FAK	(56)
circ-LRIG3	hsa_circ_0027345	chr12:59277301-59308117	1080	up	promotes proliferation, invasion, migration, inhibits apoptosis	circ-LRIG3/EZH2/STAT3	(57)
circRHOT1	hsa_circ_0005397	chr17:30500849-30503232	233	up	promotes proliferation, invasion, migration, inhibits apoptosis	circRHOT1/NR2F6	(58)
circβ-catenin	hsa_circ-0004194	chr3:41265511-41268843	1129	up	promotes proliferation, invasion, migration, inhibits apoptosis	GSK3β/Wnt/β-catenin	(59)
circDLC1	hsa_circ_0135718	chr8:12945995-12948941	552	down	inhibits migration	KIAA1429/DHX9/HuR/MMP1	(60)
cSMARCA5	hsa_circ_0001445	chr4:144464661-144465125	269	down	inhibits proliferation, migration	miR-17-3p/miR-181b-5p/TIMP3	(61)
circARSP91	hsa_circ_0085154	chr8:101721360-101721451	91	down	inhibits colony formation and tumor growth	AR/ADAR1/circRNAARSP91	(62)
circ-ADD3	hsa_circ_0020007	chr10:111876016-111886261	1274	down	inhibits migration	CDK1/EZH2	(63)
circ-102,166	hsa_circ_0004913	chr17:62248459-62265775	495	down	inhibits proliferation, invasion	miR-182/miR-184/FOXO3a/MTSS1/SOX7	(64)
circMTO1	hsa_circ_0007874	chr6:74175931-74176329	318	down	inhibits proliferation, invasion, induces apoptosis	miR-9/p21	(65)
circNFATC3	hsa_circ_0000711	chr16:68155889-68160513	1298	down	inhibits proliferation, invasion, migration, induces apoptosis	miR-548I/JNK/c-jun/AKT/mTOR	(66)
hsa_circ_0091570	hsa_circ_0091570	chrX:131516205-131526362	711	down	inhibits proliferation, invasion, migration, induces apoptosis	miR-1307/ISM1	(67)
circ ADAMTS13	hsa_circ_0089372	chr9:136302868-136303486	270	down	inhibits proliferation, induces apoptosis	miR-484	(68)
circADAMTS14	hsa_circ_0018665	chr10:72468343-72496549	929	down	inhibits proliferation, invasion, migration, induces apoptosis	miR-572/RCAN1	(69)
circZKSCAN1	hsa_circ_0001727	chr7:99621041-99621930	668	down	inhibits proliferation, invasion, migration, stemness	FMRP/CCAR1/Wnt/β-catenin	(70, 71)
circ-0051443	hsa_circ_0051443	chr19:45667421-45668228	202	down	induces apoptosis, controls cell cycle	miR-331-3p/BAK1	(72)
circ-0004277	hsa_circ_0004277	chr10:1125950-1126416	161	up	induces EMT	HuR/ZO-1	(73)
circ-0003998	hsa_circ_0003998	chr20:47570092-47580435	304	up	induces EMT	miRNA-143-3p/FOSL2	(74)
circ10720	hsa_circ_0018189	chr10:35321362-35338693	747	up	promotes proliferation, invasion, migration, induces EMT	Twist1/circ10720/miRNA-490-5P/Vimentin	(75)
circRNA-5692	hsa_circ_0005692	chr16:4382215-4383520	411	down	inhibits EMT	miRNA-328-5p/DAB2IP	(76)
circPTK2	hsa_circ_0008305	chr8:141799572-141840625	584	down	inhibits EMT	miR-92a/E-cadherin	(77)
circUHRF1	hsa_circ_0048677	chr19:4941539-4945977	625	up	mediates immune escape	miR-449c-5p/TIM-3	(78)
circMET	hsa_circ_0082002	chr7:116339124-116340338	1214	up	induces EMT, participates in immune regulation	miR-30-5p/Snail/DPP4	(79)
circTRIM 33-12	/	/	/	down	inhibits proliferation, metastasis, mediates immune escape	miR-191/TET1	(80)
hsa_circ_0007456	hsa_circ_0007456	chr17:11984672-12016677	595	down	mediates immune escape	miR-6852-3p/ICAM-1	(81)
circARNT2	hsa_circ_0104670	chr15:80767350-80772264	4914	up	enhances drug resistance	miR-155-5p/PDK1	(82)
circRNA-SORE	hsa_circ_0087293	chr9:82267504-82268990	222	up	enhances drug resistance	YBX1/AKT/Raf1/ERK/c-Myc/TGF-β,miR-103a-2-5p/miR-660-3p/Wnt/β-catenin	(83, 84)
circFN1	hsa_circ_0058124	chr2:216270960-216274462	864	up	enhances drug resistance	miR-1205/E2F1	(85)
circ_0005075	hsa_circ_0005075	chr1:21377358-21415706	205	up	promotes proliferation, invasion, migration, inhibits apoptosis, induces drug resistance	miR-335/MAPK1	(86)
circMA T2B	hsa_circ_0074854	chr5:162940560-162944680	576	up	promotes metabolic reprogramming	miR-338-3p/PI3K/AKT/mTOR/PKM2	(87)
circ-MALAT1	hsa_circ_0002082	chr11:65271199-65272066	867	up	enhances stemness	miR-6887-3p/JAK2/STAT3/PAX5	(88)
circZNF609	hsa_circ_0000615	chr15:64791491-64792365	874	up	enhances proliferation, metastasis, stemness	miR-15a-5p/15b-5p/GLI2	(89)

### Regulation of Tumorigenesis

Tumorigenesis is a progressive process. Normal cells gradually transform into tumor cells, involving the accumulation of multiple cascades and genetic mutations, among which circRNAs play a crucial role.

E1A binding protein p300 (EP300) and WD repeat domain 5 (WDR5) are recruited to the circSOD2 promoter, stimulating the H3K27ac and H3K4me3-mediated modification to upregulate circSOD2, which further acts as a molecular sponge to inhibit the expression of miR-502-5p, in order to mediate the overexpression of its downstream target gene DNA methyltransferase 3 alpha (DNMT3a). Highly expressed DNMT3a reduces the expression of suppressor of cytokine signaling 3 (SOCS3) by promoting the over-methylation of CpG islands in the SOCS3 promoter, thereby activating the Janus kinase 2/signal transducer and activator of transcription 3 (JAK2/STAT3) signaling pathway to drive the tumorigenesis of HCC. Meanwhile, STAT3 can combine with the circSOD2 promoter to form a positive feedback loop, persistently activating its transcription and maintaining a high-expression status (32). Hepatocyte nuclear factor 4 alpha (HNF4a) can bind to the circ_104075 promoter to activate its transcription, then upregulating YAP through sponge miR-582-3p to advance the process of HCC (33). circTMEM45A relieves the inhibition of insulin like growth factor 2 (IGF2) expression by interacting with miR-665 to promote HCC tumorigenesis (34). Knocking down circ_0001955 can significantly suppress the proliferation of HCC cells and subcutaneous xenografts growth *in vivo*, showing a smaller size and weight. Circ_0001955 can directly bind to miR-516a-5p as a competing endogenous RNA (ceRNA), which further mediates the upregulation of oncogenes TNF receptor associated factor 6 (TRAF6) and mitogen-activated protein kinase 11 (MAPK11) (35). Hsa_circ_0016788 also promotes the tumorigenesis of HCC through hsa_circ_0016788/miR-486/cyclin dependent kinase 4 (CDK4) axis (36). Functional assays revealed that overexpressed circSETD3 can restrain the proliferative capacity of HCC cells, as well as inducing G1/S phase arrest *in vitro*. Simultaneously, circSETD3 knockdown could effectively accelerate the growth rate of subcutaneous xenografts *in vivo*. CircSETD3 suppress the tumorigenesis of HCC through the circSETD3/miR-421/mitogen-activated protein kinase 14 (MAPK14) pathway (37).

### Regulation of Malignant Biological Phenotypes

Affected by continuous proliferation signals, tumor cells with infinite growth potential can escape programmed cell apoptosis, accelerate the cell cycle process to achieve the long-term survival. Next, tumor cells originating from the primary tissues colonize other sites through body cavities, blood vessels, or lymphatic tracts, forming metastatic lesions. It is reported that circRNAs play an important regulatory role in the malignant biological behaviors of HCC cells.

Through binding to the 3’untranslated region (UTR) of stearoyl-CoA desaturase (SCD) mRNA, RNA binding motif protein 3 (RBM3) increases the production of SCD-circRNA 2, which enhances the phosphorylation of extracellular regulated protein kinase (ERK) and promotes the proliferation of HCC cells (38). Overexpressed circBACH1 activates the transport of human antigen R (HuR) from the nucleus to the cytoplasm, inhibiting p27 translation by abolishing the internal ribosome entry site (IRES) located at the 5’-UTR of p27, thereby promoting the proliferation of HCC cells and accelerating the cell cycle process (39). Has_circ_0000204 can sponge miR-191 to upregulate downstream target kruppel like factor 6 (KLF6), further promoting HCC cell proliferation and cell cycle transition (40). Exogenous knockdown of exosomal circRNA-100,338 can inhibit the invasive ability of HCC cells and the growth rate of xenografts, reducing the microvessel density in tumors and the number of lung metastatic nodules. Transported to human umbilical vein endothelial cells (HUVECs), exosomal circRNA-100,338 can stimulate the cell proliferation, and destroy the tight junctions between HUEVCs to affect their permeability. Meanwhile, the interaction with NOVA alternative splicing regulator 2 (NOVA2) induces angiogenesis and transfer ability of HCC cells (41). Enriched in HCC cell-derived exosomes characterized by high metastatic potential, circPTGR1 can upregulate MET proto-oncogene (MET) by competitively binding miR-449a, activating the metastatic activity of HCC cells with low or no metastatic potential, thereby destroying the tumor microenvironment homeostasis and promoting the invasion and metastasis of HCC cells (42). Silencing circASAP1 can restrain the proliferation, migration, and invasion of HCC cells, as well as impeding the growth and lung metastasis rate of xenografts. circASAP1 stimulates the expression of mitogen-activated protein kinase 1 (MAPK1) through sponging miR-326 and miR-532-5p, which further activates the ERK1/2 signaling pathway to promote the proliferation and invasion of HCC cells; additionally, MAPK1 de-inhibits colony stimulating factor 1 (CSF-1) and promotes the proliferation and chemotactic migration of tumor-associated macrophages (TAMs), mediating TAM infiltration into the tumor bed, which is benefit for the tumor metastasis (43). Through directly sponging miR-1299, circMAST1 rescues the expression suppression of catenin delta 1 (CTNND1), inducing the proliferation and invasion of HCC cells. Silencing circMAST1 reduces the growth rate of xenografts, while the expression levels of proliferating cell nuclear antigen (PCNA) protein and cell cycle-related proteins such as cyclin A, cyclin E, CDK1, and CDK2 are significantly decreased (44). CircDYNC1H1 negatively regulates the expression of miR-140-5p, relieving the expression inhibition of sulfotransferase family 2B member 1 (SULT2B1) mediated by miR-140-5p, further enhancing the proliferative and metastatic capacity of HCC cells (45). Highly expressed circSLC3A2 promotes the proliferation and invasion of HCC cells by circSLC3A2/miR-490-3p/protein phosphatase, Mg2+/Mn2+ dependent 1F (PPM1F) axis (36). CircHIPK3 acts as a miR-124 sponge to regulate its downstream target gene aquaporin 3 (AQP3), promoting the proliferation and metastasis of HCC cells, while silencing circHIPK3 inhibits the proliferation and migration of HCC cells *in vitro* and delaying the growth rate of subcutaneous xenografts *in vivo* (47). Hsa_circ_0001955 promotes the proliferation, invasion, and migration of HCC tumor cells through the miR-145-5p/NRAS pro-oncogene (NRAS) axis (48). Exogenous silencing circ-TCF4.85 can effectively inhibit the proliferation, invasion, and migration of HCC cells, as well as blocking the cell cycle process (49). Through competitively binding miR-4738-3p, circZNF566 blocks the direct interaction of miR-4738-3p and the 3’-UTR of tryptophan 2,3-dioxygenase (TDO2) mRNA, which further leads to the upregulation of TDO2, thereby promoting the proliferation, invasion, and metastasis of HCC cells, while cancer-promoting effect can be reversed by the knockdown of circZNF566 (50). Hsa_circRNA 103809 directly binds to miR-377-3p and negatively regulates its expression, releasing the inhibition of fibroblast growth factor receptor 1 (FGFR1), and then promotes the proliferation, metastasis, and cell cycle progression of HCC cells (51). Hsa_circ_104348 can act as a ceRNA of miR-187-3p, blocking its binding to the downstream target gene rhotekin 2 (RTKN2) to achieve the same trend expression of RTKN2 and hsa_circ_104348, and further activating the Wnt/β-catenin pathway, thereby affecting tumor cell proliferation, invasion, metastasis, and anti-apoptosis ability (52). Analogously, circRNA-104718 can also act as a ceRNA to directly bind to miR-218-5p, reducing the inhibition mediated by miR-218-5p upon its target gene thioredoxin domain containing 5 (TXNDC5), thereby promoting the growth and metastasis of HCC (53). CircFBLIM1 can acts as a miR-346 sponge through the ceRNA mechanism to regulate the expression of filamin binding LIM protein 1 (FBLIM1) and promote the progress of HCC (54). The expression of exosomal circ-DB is significantly upregulated in HCC patients with higher body fat ratio. The adipocyte-derived exosomes of HCC patients act as carriers of circ-DB, promoting the growth and reducing DNA damage *via* the suppression of miR-34a and the activation of deubiquitination-related ubiquitin specific peptidase (USP7) (55).

Overexpressed circRASGRF2 promotes the malignant biological behaviors of HCC through the circRASGRF2/miR-1224/focal adhesion kinase (FAK) signal axis. Moreover, knocking down circRASGRF2 can effectively suppress the proliferation, invasion, and migration of HCC tumor cells, and induce cell cycle arrest and apoptosis, as well as significantly slowing down the growth rate of xenografts in nude mice and inhibit the lung metastasis effect of tumors (56). Circ-LRIG3 can interact with enhancer of zeste homolog 2 (EZH2) and STAT3, acting as a scaffold to increase STAT3 methylation and subsequent phosphorylation induced by EZH2. Activated STAT3 can directly bind to the circ-LRIG3 promoter to enhance the transcriptional activity of circ-LRIG3, and then forms positive feedback loop to promote the progress of HCC (57). CircRHOT1 recruits Tat interactive protein 60 (Tip60) to the nuclear receptor subfamily 2 group F member 6 (NR2F6) promoter, subsequently recruiting NuA4 complex-related components to stimulate the expression of NR2F6. Meanwhile, the circRHOT1/Tip60/NR2F6 axis may partially activate the notch receptor 2 (NOTCH2) signaling pathway (58). β-catenin-370aa competitively interacts with glycogen synthase kinase 3β (GSK3β) and acts as a decoy, antagonizing GSK3β-induced β-catenin phosphorylation and degradation to stabilize full-length β-catenin, thereby activating the Wnt/β-catenin pathway to promote the malignant phenotypes of HCC (59). CircRNAs can also act as a tumor suppressor to inhibit the malignant biological behaviors of HCC. As a novel downstream effect target of m6A modification mediated by KIAA1429, the expression of circDLC1 in HCC can be inhibited by DExH-box helicase 9 (DHX9). Through interacting with HuR, circDLC1 reduces the stability of matrix metallopeptidase 1 (MMP1) mRNA and downregulates its expression to restrain HCC metastasis (60). DHX9 can also downregulate cSMARCA5, which promotes the expression of tissue inhibitor of metalloproteinase 3 (TIMP3) by sponging miR-17-3p (61). Androgen receptor (AR) suppresses the expression of circARSP91 by stimulating adenosine deaminase acting on RNA 1 (ADAR1) in combination with the ADAR1 promoter. CircARSP9 can act as a tumor suppressor in HCC to inhibit tumor growth (62). Circ-ADD3 can enhance the interaction between CDK1 and EZH2, mediating the increase of EZH2 ubiquitination, which mediates the degradation of EZH2. Reduced EZH2 significantly increases the expression of a series of antimetastatic genes, including circ-ADD3, by reducing the level of H3K27me3 in the promoter region to form a regulatory circuit, thereby inhibiting the metastasis of HCC (63). By releasing the tumor-suppressor genes miR-182 and miR-184, circ-102 and circ-166 reduce the expression of downstream target genes FOXO3a, MTSS I-BAR domain containing 1 (MTSS1), and SRY-box transcription factor 7 (SOX7), as well as increasing the levels of c-myc protein and Rb phosphorylation, thereby inhibiting the proliferation and invasion of HCC cells (64). CircMTO1 promotes the expression of p21 by acting as a sponge for the oncogene miR-9 to inhibit the proliferation and invasion of HCC. Knocking down circMTO1 can effectively promote the proliferation and invasion of HCC cells and inhibit cell apoptosis. Meanwhile, the growth rate of xenografts in the knockdown group also significantly accelerates (65). CircNFATC3 acts as a ceRNA combined with miR-548 to protect the maternal gene nuclear factor of activated T cells 3 (NFATc3). NFATc3 and circNFATC3 can synergistically interfere with the phosphorylation of the c-Jun NK2-terminal kinase (JNK)/c-jun/serine/threonine kinase (AKT)/mechanistic target of rapamycin kinase (mTOR) cascade to inhibit the progression of HCC, while overexpressed circNFATC3 can inhibit the proliferation of HCC cells, inducing cell apoptosis and weakening the ability of invasion and migration; meanwhile, the size and weight of xenografts in the overexpression group were significantly reduced than those in the knockdown group, and the lung metastasis effect was inhibited (66). Hsa_circ_0091570 can also act as a ceRNA to bind miR-1307 and upregulate the expression of isthmin 1 (ISM1) to inhibit tumor progression (67). Circ ADAMTS13 can sponge miR-484 to inhibit the proliferation of HCC cells and induce apoptosis (68). CircADAMTS141 can competitively bind to miR-572 and inhibit its transcriptional activity, thereby promoting the expression of the downstream target gene regulator of calcineurin 1 (RCAN1) (69). Kyoto encyclopedia of genes and genomes (KEGG) enrichment analysis showed that after knocking down cirZKSCAN1, differentially expressed genes are more likely to be enriched in phosphatidylinositol 3-kinase (PI3K) pathway, migration pathway, actin cytoskeleton pathway, adhesion pathway, cytokine interaction pathway, and other tumor-related signal pathways. Zinc finger with KRAB and SCAN domains 1 (ZKSCAN1) mRNA regulates cell metabolism, suggesting that ZKSCAN1 mRNA and circZKSCAN1 may suppress HCC progression through interaction. The overexpression of cirZKSCAN1 can effectively inhibit cell proliferation, invasion, and migration. The xenografts in the knockdown group shows growth inhibitory effect (70). Exosomes transfer circ-0051443 from normal cells to HCC cells. Through competitive binding of miR-331-3p, circ-0051443 mediates the upregulation of downstream target gene BCL2 antagonist/killer 1 (BAK1), further promoting cell apoptosis, blocking cell cycle in G0/G1 phase, and inhibiting malignant biological behaviors of HCC cells (72).

### Regulation of Epithelial-Mesenchymal Transition Process

EMT refers to the biological process in which differentiated epithelial cells transform into cells with a mesenchymal phenotype, which endows malignant tumor cells with abilities such as invasion and migration, stem cell characteristics, and immunosuppression.

Exogenous silencing circ-0004277 inhibits HCC cell proliferation and migration, mediating smaller volume and weight and lung metastasis inhibitory effect of xenografts. Studies have shown that circ-0004277 competitively binds to HuR, blocking its binding with zonula occludens-1 (ZO-1) mRNA, downregulating ZO-1, and stimulating the EMT process. Exosome circ-0004277 derived from HCC cells can mediate the communication between surrounding normal cells and HCC cells, stimulating the EMT process of surrounding normal cells, and promote the progress of HCC invading into surrounding normal tissues (73). Overexpressed circ-0003998 can promote the proliferation of HCC cells, significantly promoting the lung metastasis, while knockdown of circ-0003998 inhibits the opposite results. Circ-0003998 can act as a ceRNA of miRNA-143-3p to impair the expression inhibition of FOS-like antigen 2 (FOSL2) (EMT-related stimulator); meanwhile, circ-0003998 can also combine with poly(rC) binding protein 1 (PCBP1) to increase the expression of CD44v6 (EMT-related genes), then promote the EMT process of HCC (74). Twist1 directly binds to the cullin 2 (Cul2) promoter to activate its transcription and selectively promotes the expression of circ10720 in Cul2. Circ10720 induces EMT in HCC cells by sponging miRNA and upregulating the expression of the target gene Vimentin (75). The overexpression of circRNA-5692 significantly increased the expression of E-cadherin, while reducing the expression of Vimentin and Snail. CircRNA-5692 restrain the proliferative and invasive ability of tumor cells, as well as inducing apoptosis. Overexpressed circRNA-5692 can serve as ceRNA to spongy miRNA-328-5p, reducing its inhibitory effect on DAB2 interacting protein (DAB2IP) expression and promoting the demethylation of DAB2IP gene to weaken the EMT process (76). Similarly, circPTK2 can be used as ceRNA to absorb miR-92a, so as to upregulate E-cadherin and inhibit the EMT process of HCC (77).

### Regulation of the Immune System

The immune escape of tumor cells is an important part of the progression of tumors. After cancer cells evade the body’s immune surveillance and attack through various mechanisms, malignant biological behaviors such as proliferation, invasion, and metastasis are further enhanced, resulting in the loss control of tumor cell growth upon body’s immune system.

CircUHRF1 was upregulated in HCC tissues and cancer cell–derived exosomes. Overexpressed plasma exosome circUHRF1 was associated with a decreased natural killer cell (NK) proportion and reduced NK cell tumor infiltration. HCC cell-derived exosomes can transfer circUHRF1 into peripheral NK cells and inhibit its activity by sponging miR-449c-5p to achieve the high expression of downstream target gene T-cell immunoglobulin mucin 3 (TIM-3), thereby inhibiting the secretion of interferon-γ (IFN-γ) and tumor necrosis factor-α (TNF-α) from NK cells, leading to impaired function and phenotypic exhaustion of NK cells to promote immune escape of HCC cells (78). Highly expressed circMET can induce EMT or degrade C-X-C motif chemokine ligand 10 (CXCL10) *via* the circMET/miR-30-5p/Snail/dipeptidyl peptidase 4 (DPP4) axis to reduce CD8+ T lymphocyte transport, thereby enhancing the formation of the immunosuppressive tumor microenvironment to promote tumor progression (79). CircTRIM 33-12 acts as ceRNA and upregulates tet methylcytosine dioxygenase 1 (TET1) expression by competitively binding miR-191, promoting the expression of tumor-suppressor genes [WWC family member 3 (WWC3), tumor protein p53 nuclear protein 1 (TP53INP1), UL16 binding protein 1 (ULBP1), jumonji C domain containing hiatone demethylase 1 homolog D (JHDM1D)] in HCC cells, and reducing 5-hydroxymethylcytosine (5hmC) content, subsequently inhibiting the proliferation and metastasis of HCC cells and inducing immune evasion (80). The expression of hsa_circ_0007456 in HCC cell lines and clinicopathological tissues was significantly downregulated, which interferes with the sensitivity of HCC cells to NK cells by reducing the binding of NK cells and tumor cells. Hsa_circ_0007456 can also interact with miRNAs and endogenously adsorb miR-6852-3p, blocking its binding to the downstream target gene intercellular adhesion molecule 1 (ICAM-1) 3’-UTR, affecting the expression of ICAM-1 and promoting the immune escape of tumor cells (81).

### Regulation of Drug Resistance

Chemotherapy is one of the main methods of tumor treatment, which can effectively reduce the recurrence and metastasis of tumors. While molecular targeting chemotherapy have recently experienced rapid progress, the existence of chemotherapy resistance still limits the advancement of long-term survival. Therefore, understanding the underlying molecular mechanism of HCC chemoresistance and developing mechanism-based therapies are urgently needed. CircRNA has been shown to be involved in the development of drug resistance.

CircARNT2 is upregulated in HCC tissues, cell lines, and cisplatin-resistant cells, and knocking down circARNT2 can significantly inhibit tumor cell proliferation and aggravate cisplatin-induced apoptosis. CircARNT2 can act as ceRNA to competitively bind miR-155-5p, regulating the autophagy induced by pyruvate dehydrogenase kinase 1 (PDK1) and then promoting the cisplatin resistance of HCC cells (82). circRNA-SORE is upregulated in sorafenib-resistant HCC cells. Knockdown of circRNA-SORE can significantly enhance the cytotoxicity of sorafenib, leading to cell morphology destruction and increased apoptosis. CircRNA-SORE binds to Y-box binding protein 1 (YBX1) in the cytoplasm to prevent it from translocating to the nucleus, thereby inhibiting PRP19-mediated ubiquitination and degradation of YBX1, affecting the expression of YBX1 downstream gene targets AKT, Raf1, ERK, c-Myc, and TGF-β1, thereby inducing the sorafenib resistance. CircRNA-SORE can also achieve the diffusion of sorafenib-resistant in HCC cells through the enrichment and transferation of HCC cell-derived exosomes (83). The increase in the expression level of m6A at the specific binding site of circRNA-SORE increases the stability of RNA, which in turn upregulates the expression level of circRNA-SORE in HCC sorafenib-resistant cells. The highly expressed circRNA-SORE acts as ceRNA to specifically sponge miR-103a-2-5p and miR-660-3p, activating Wnt/β-catenin pathway to induce sorafenib-resistant (84). Overexpression of circFN1 can positively regulate the expression of E2F transcription factor 1 (E2F1) by interacting with miR-1205 to achieve sorafenib-resistance in HCC cells, whereas silencing circFN1 can promote the expression of phosphatase and tensin homolog (PTEN) protein and inhibit the activation of AKT in HCC cells, enhancing sorafenib sensitivity in HCC cells (85). Circ_0005075 can bind to miR-335 and antagonize the inhibitory effect of miR-335 on the downstream target gene MAPK1, thereby enhancing the proliferation, migration, invasion, and anti-apoptotic abilities of HCC cells (86).

### Regulation of Metabolic Reprogramming

Tumor cells adjust glucose metabolism from oxidative phosphorylation to glycolysis through metabolic reprogramming to adapt to hypoxic stress. Even in the case of sufficient oxygen supply, cancer cells still preferentially use glycolysis instead of the tricarboxylic acid cycle pathway of mitochondrial to decompose glucose, providing ATP and glycolysis intermediates for the metabolism and biosynthesis of cancer cells, as well as developing a tumor microenvironment suitable for cancer cells to survive, thus avoiding the immune and apoptotic procedures of the body, creating advantages for tumor cells to proliferate and metastasis (90).

*In vivo* and *in vitro* functional experiments have shown that siRNA-circMA T2B can inhibit the glycolysis capacity and rate of HCC cells, forming a low glucose uptake, low lactate production status, and reduced ATP levels of tumor cells. Meanwhile, it also activates mitochondrial oxidative phosphorylation, causing higher oxygen consumption of cells in the basal and maximum respiration state; under hypoxic stress, siRNA-circMA T2B can significantly suppress the proliferation, invasion, and migration of HCC cells and induce increased apoptosis. In the knockdown group, xenografts exhibit glycolysis, growth, and lung metastasis inhibitory effects, showing a lower level of glycolysis-related organic acids, a slower growth rate, and fewer lung metastasis nodules. Highly expressed circMA T2B acts as a sponge of miR-338-3p and inhibits its transcriptional activity, thereby increasing the expression of the target gene kinesin family member C1 (KIFC1), activating the PI3K/AKT/mTOR signaling pathway and upregulating the expression of pyruvate kinase M2 (PKM2), subsequently promoting glycolysis and malignant phenotypes of HCC cells under hypoxic conditions (87).

### Regulation of Cellular Stemness

Cancer stem cells (CSCs) have the ability to self-renew and differentiate into different cell types. They can participate in the mediation of tumor initiation, metastasis, chemotherapy resistance, and recurrence, closely related to poor prognosis (91).

Under the mediation of AU-rich element RNA-binding factor 1 (AUF1), circ-MALAT1 was upregulated in HCC CSCs. Highly expressed circMALAT1 reduces the inhibitory effect of miR-6887-3p on JAK2 through molecular sponge action, thereby upregulating the expression levels of JAK2 and enhancing its phosphorylation, then activating the JAK2/STAT3 signaling pathway to promote the self-renewal of HCC CSCs; meanwhile, circMALAT1 can also combine with ribosomal and paired box 5 (PAX5) mRNA coding sequences to form a specific ternary complex (ribosome-circRNA-mRNA) to exert mRNA braking. It directly hinders the translation of PAX5 mRNA and affects PAX5-related cell functions to promote the self-renewal of HCC CSCs (88). The expression of circZNF609 is higher in HCC tissues than in normal tissues. Knocking down circZNF609 represses the expression level of transfer-related proteins [matrix metalloproteinase (MMP2), (MMP7)], stemness-related transcription factors (OCT4 and Nanog), EMT-related proteins (N-cadherin, Twist), while the proliferation and spheroidizing ability of HCC cells are significantly inhibited. Further studies have shown that circZNF609 activates the Hedgehog pathway by inhibiting the expression of miR-15a-5p/15b-5p and upregulating the expression of GLI family zinc finger 2 (GLI2), a downstream target of miR-15a-5p/15b-5p, thereby enhancing proliferation, metastasis, and stemness of HCC cells (89). circZKSCAN1 is downregulated by Quaking (QKI) in HCC. CircZKSCAN1 blocks the binding of fragile X mental retardation protein (FMRP) to cell division cycle and apoptosis regulator 1 (CCAR1) mRNA by competitively binding to FMRP, and subsequently inhibits the transcriptional activity of the Wnt/β-catenin signaling pathway, thereby inhibiting the malignant biology behaviors of HCC cells by regulating the stemness of HCC cells (71).

### CircRNAs as Indicators for Patient Outcomes

Dysregulated expression of circRNAs is closely related to the clinicopathological characteristics of HCC patients. Accurately predicting the clinical prognosis of HCC patients can help guide decision-making in HCC treatment, thereby effectively improving the survival benefits of patients. At the same time, due to the high abundance and high stability in HCC clinical samples, circRNA can be used as a potentially effective biomarker for the clinical prognosis of HCC patients.

Nine circRNAs, were greatly upregulated in HCC tissues, namely, circARNT2, circRASGRF2, circFN1, circRNA-104718, circSLC3A2, circ-10720, circSOD2, circ_0005075, and circRHOT1 (32, 46, 53, 56, 58, 75, 82, 85, 86). All these highly expressed circRNAs have been confirmed to be potential prognostic biomarkers for poor outcome in HCC by Kaplan-Meier (KM) analysis. Clinically, the expression of circARNT2 was closely related to tumor size, TNM stages, and distant metastasis (82), while the expression of circFN1 was correlated with HCC tumor size, TNM stages, and vascular infiltration (85). Higher expression of circRASGRF2 in HCC tissues indicates poor tumor differentiation, later tumor stages, larger tumor size (>5 cm), and the presence of microvascular infiltration (MVI) (56). circRNA-104718 expression was correlated with vascular infiltration (53); circ-10720 expression was related to serum AFP level and hepatitis B markers (75). Increased circSOD2 expression was associated with higher grade tumors (32). High expression of circRNAs not only in HCC tissues but also in HCC cell lines was also identified to be potential prognostic biomarkers for poor OS, circZNF566, has_circ_104348, circRNA_103809, and circBACH1, respectively (39, 50–52); simultaneously, highly expressed circZNF566 was negatively correlated with disease-free survival (DFS) (50). In terms of clinicopathological characteristics, upregulated circZNF566 was positively correlated with tumor size, tumor differentiation, and M stage (50). The expression of has_circ_104348 was further correlated with tumor size, lymph node invasion, and TNM stages (52), while circBACH1expression was associated with tumor size and histological differentiation (39). In other studies, higher expression of four circRNAs could predict poor prognosis of HCC patients by KM analysis, including circRNA-SORE, circMET, SCD-circRNA 2, circASAP1 (38, 43, 79, 84); in addition, HCC patients with high expression of circRNA-SORE have poor recurrence-free survival (RFS), so did SCD-circRNA 2. Moreover, patients in the high-expression group of circMET have higher cumulative recurrence rates (38, 79, 84). Clinically, circMET expression was closely correlated with MVI, multiple tumors, the presence or absence of tumor envelope, as well as advanced stages, while SCD-circRNA 2 expression was associated with serum AFP levels, alanine aminotransferase (ALT) and aspartate aminotransferase (AST) levels. Besides, circHIPK3 was reported to be upregulated in HCC tissues, and its expression was related to the degree of tumor differentiation, TNM stages, HBV-DNA replication, and the presence of LC (47). High circ0003998 expression has been observed in HCC tissues, which is also correlated with advanced TNM stages and high level of serum AFP (74). The expression of hsa_circ_0001955 in HCC tissues and cell lines was significantly elevated and positively correlated with larger tumor size and advanced TNM stages (48).

Except for HCC tissues and cell lines, several circRNAs upregulated in plasma, serum, and exosomes (serum-derived or cancer cell-derived) were identified as potent non-invasive prognostic biomarkers by KM analysis, such as circTMEM45A, circ-LRIG3, circUHRF1, circRNA-100,338, circPTGR1 (34, 41, 42, 57, 78). Among them, circTMEM45A expression was remarkably correlated with tumor size, TNM stages, vascular infiltration, and survival time of HCC patients (34). Patients in the high-expression group of circLRIG3 have larger tumor size, more vascular invasion, higher Edmondson’s grade, TNM stages, and shorter OS and DFS. Multivariate analyses revealed that the expression of circLRIG3 may be used as an independent risk prognostic assessment factor (57). Higher expression of circUHRF1 was shown to be associated with larger tumor size, fewer NK cells in the blood, and more capillaries infiltration, and worse clinical prognosis. Moreover, circUHRF1 was confirmed to be an independent indicators of OS and postoperative recurrence in the disease by multivariable analyses (78). Interestingly, univariate and multivariate analyses demonstrated that the continuous high expression of circRNA-100,338 in the serum of HCC patients undergoing radical hepatectomy may serve as a risk factor of lung metastasis and poor prognosis predictor (41). Similarly, circMA T2B was identified to be independent prognostic indicators for HCC patients by multivariable analyses. In addition, circMA T2B expression was related to tumor size, vascular infiltration, tumor multiplicity, tumor envelope, lymph node metastasis, as well as Edmonson stage (87).

Accumulating evidence has certificated that compared with these upregulated circRNAs, lower expression of other circRNAs could predict poor outcome of HCC patients. For instance, 12 circRNAs—namely, circDLC1, circ-102,166, circ-ADD3, circADAMTS13, circZKSCAN1, cSMARCA5, hsa_circ_0091570, circSETD3, circNFATC3, circTRIM33-12, hsa_circ_0007456, circMTO1—were significantly downregulated in HCC tissues and cell lines (37, 60, 61, 63–68, 70, 80, 81). Kaplan-Meier (KM) analysis demonstrated that lower expression of all these 12 circRNAs was associated with poor OS of HCC. Moreover, the first six lessened expression of circRNAs were correlated with shorter RFS of HCC patients as well. Of course, downregulated circRNAs were also associated with the clinicopathological characteristics of HCC patients. The low expression of circRNA-5692 was closely correlated with abnormally high levels of AFP, history of LC, larger tumor size, and distant metastasis (76). Other research shows that the expression of circ-102,166 significantly correlated with tumor size, TNM stages, Barcelona Clinic liver cancer (BCLC) stages, and vascular infiltration (64); lower expression of circ-ADD3 was correlated with vascular infiltration, intrahepatic metastasis, distant metastasis, and progression-free survival (PFS) (63). Lower expression of hsa_circ_0091570 was related to Edmondson Grade, portal vein tumor thrombus (67), while lower expression of circSETD3 was significantly associated with larger tumor size, poor tumor differentiation (37). Lower expression of circADAMTS13 was associated with the absence of LC, larger tumor size, and advanced BCLC stages (68). circZKSCAN1 was related with number of tumors, LC, tumor grade, and MVI (70, 71). Reduced expression of circTRIM33-12 was related to larger tumor size, multiple tumors, encapsulation invasion, and MVI, as well as elevated AFP levels (80), while decreased expression of cSMARCA5 is closely related to poor tumor differentiation, advanced tumor stage, larger tumor size, and MVI. Interestingly, multivariate analyses demonstrated that the expression of circTRIM33-12 and cSMARCA5 may serve as independent prognostic evaluation index for HCC patients (61). Similarly, multivariate analyses indicated that low circDLC1 and circNFATC3 expression in HCC tissues can be used as independent risk factors of poor prognosis for HCC patients (60, 66).

### CircRNAs as Diagnostic Biomarkers for HCC

Insidious onset and lack of accurate and effective biomarkers for early diagnosis of HCC are the main reasons for low OS in HCC patients. Traditional diagnostic markers, such as AFP, AFP-L3, α-L-fucosidase (AFU), and protein induced by vitamin K absence or antagonist-II (PIVKA-II), have low sensitivity and specificity for the diagnosis of HCC. Studies have shown that lncRNAs and miRNAs have been reported as potential biomarkers for the diagnosis of HCC. Based on the high abundance of circRNA in HCC tissues, body fluids, and exosomes, the resistance to ribonuclease, and the highly conservative and specific expression of evolution, circRNA can be used as an ideal biomarker for the early diagnosis of HCC **(** **Table 2** **)**.

**Table 2:** CircRNAs as diagnostic biomarkers for HCC.

CircRNA	Expression	Samples	AUC	Sensitivity	Specificity	Youden index (YI)	Confidence interval (CI)	References
circRASGRF2	up	tissues	0.88	/	/	/	0.814–0.950	(56)
circBACH1	up	tissues	0.85	/	/	/	/	(39)
circFN1	up	tissues	0.88	/	/	/	0.794–0.963	(85)
circ-LRIG3	up	tissues	0.87	/	/	/	0.7843–0.9519	(57)
circTCF4.85	up	tissues	0.89	0.868	0.870	0.738	0.820–0.962	(49)
hsa_circ_0016788	up	tissues	0.85	/	/	/	/	(36)
hsa_circ_0091570	down	tissues	0.74	/	/	/	/	(67)
circZKSCAN1	down	tissues	0.83	0.822	0.724	0.546	/	(60)
circADAMTS13	down	tissues	0.99	/	/	/	/	(68)
circ_104075	up	tissues, serum	0.97	0.969	0.983	0.943	0.780–0.995	(33)
circ-0004277	up	serum exosomes	0.82	0.583	0.967	0.55	0.741–0.891	(73)
circTMEM45A	up	serum exosomes	0.89	/	/	/	0.823–0.954	(34)
circ-0051443	down	tissues, plasma	0.81	/	/	/	/	(72)
circ-ADD3	down	tissues, plasma	0.89	/	/	/	0.8094–0.9662	(63)
cSMARCA5	down	plasma	0.94	0.867	0.893	0.76	0.910–0.966	(92)
hsa_circ_0005075	up	tissues	0.94	0.833	0.9	0.733	/	(93)
hsa_circ_0128298	up	tissues	0.67	0.674	0.805	0.479	0.503–0.794	(94)
circ-CDYL	up	tissues	0.64	0.333	0.928	0.261	0.55–0.72	(95)
hsa_circ_0004018	down	tissues	0.85	0.716	0.815	0.531	0.803–0.894	(97)
hsa_circ_000244	up	tissues, serum	0.97	0.956	0.927	0.883	0.948–0999	(97)
hsa_circ_000520	down	tissues, serum	0.94	0.971	0.896	0.876	0.902–0.984	(97)
hsa_circ_001565	down	tissues, serum	0.84	0.735	0.823	0.555	0.771–0.907	(97)

In HCC tissues, nine upregulated circRNAs, namely, circRASGRF2, circBACH1, circFN1, circ-LRIG3, circTCF4.85, hsa_circ_0016788, hsa_circ_0005075, hsa_circ_0128298, circ-CDYL (36, 39, 49, 56, 57, 85, 93–95), constitute potential diagnostic biomarkers in HCC. Among them, the first seven circRNAs reached higher area under the receiver operating characteristic curve (AUC) value of 0.882, 0.8506, 0.878, 0.8681, 0.891, 0.851, 0.94, respectively. At the same time, the sensitivity of circTCF4.85 to distinguish HCC patients from healthy controls was 86.8%, and the specificity was 87.0%, and the sensitivity and specificity of hsa_circ_0005075 were 83.3 and 90.0% (48, 93). In contrast, the upregulation of hsa_circ_0128298 (AUC value: 0.668, sensitivity: 0.674; specificity: 0.805) and circ-CDYL (AUC value: 0.64, sensitivity: 0.333; specificity: 0.928) demonstrated relatively poorer diagnostic value (94, 95). The downregulated expression of circRNAs in HCC tissues also has high diagnostic value. The AUC values of hsa_circ_0091570, circZKSCAN1, circADAMTS13, and hsa_circ_0004018 are 0.736, 0.834, 0.987, 0.848, respectively (50, 67, 68, 96). Among them, the sensitivity and the specificity of circZKSCAN1 as a diagnostic biomarker for HCC were 82.2 and 72.4%, as well as the sensitivity and the specificity of hsa_circ_0004018 were 71.6 and 81.5% (70, 96).

The expression of circRNAs was dysregulated not only in HCC tissues but also in serum, plasma, and exosomes, indicating that it may be used as a non-invasive circulating biomarker for HCC diagnosis. Study has shown that the expression levels of hsa_circ_000244 (AUC value: 0.974, sensitivity: 0.956; specificity: 0.927) and circ_104075 (AUC value: 0.973, sensitivity: 0.969; specificity: 0.983) in HCC tissues and serum were significantly higher than that of healthy individuals and showed a significant diagnostic value (33, 97). While four others circRNAs, namely, circ-0051443, circ-ADD3, cSMARCA5, hsa_circ_000520, and hsa_circ_001565, were reported to be downregulated in HCC tissues and serum/plasma of HCC patients and achieved diagnostic potential with AUC values of 0.8089, 0.8878, 0.938, 0.943, and 0.839, respectively (63, 72, 92, 97). Among them, the sensitivities of hsa_circ_000520 and hsa_circ_001565 as diagnostic biomarkers were 97.1 and 73.5%, the specificities were 89.6 and 82.3%, respectively. The sensitivity and specificity of cSMARCA5 were 86.7 and 89.3% (92, 97). In addition to the above circRNAs, dysregulated circRNAs in exosomes of HCC patients have potential diagnostic value as well. For example, upregulated exosomes circ-0004277 and exosomes circTMEM45A reached potent AUC values of 0.816, 0.818, respectively (34, 73). Moreover, the sensitivity and specificity of circ-0004277 used to distinguish HCC patients from healthy controls are 58.3 and 96.7%.

Combined detection can significantly improve the accuracy of HCC diagnosis. By a microarray screening and quantitative real-time polymerase chain reaction (qRT-PCR) in a multicenter study, a circPanel containing three HBV-related HCC plasma upregulated expressions of circRNA (hsa_circ_0000976, hsa_circ_0007750, and hsa_circ_0139897) was identified. circPanel is superior to AFP in the diagnosis of HCC and small HCC. It can also effectively identify AFP-negative HCC and AFP-negative small HCC (AUC are greater than 0.80) (98). Studies have shown that the expression of circ_0009582, circ_0037120, and circ_0140117 in HBV-related HCC tissues is significantly higher than that in chronic hepatitis and healthy subjects. The combined detection of these three circRNA and alpha-fetoprotein has higher sensitivity and specificity (99).

## Conclusion

HCC is one of the most malignant tumors, and the high mortality rate makes it urgent to develop effective tools for early diagnosis and clinical treatment. Emerging evidence reveals that the dysregulated circRNA expression in HCC clinical specimens was closely related to the clinicopathological characteristics of HCC patients and can act as a miRNA sponge, interact with RBP or a transcriptional regulator, and then participate in the regulation of the HCC cells tumorigenesis, proliferation and anti-apoptosis, invasion and metastasis, EMT, immune escape, drug resistance, metabolic reprogramming, and other biological processes. So, targeting of circRNA in HCC patients may reverse the progress of HCC, so as to develop new therapeutic strategies for HCC. For example, targeting of circRNA-SORE in sorafenib-treated HCC patients as a novel targeted therapy for advanced HCC, circRNA-SORE can sequester miR-103a-2-5p and miR-660-3p by acting as a miRNA sponge, thereby competitively activating the Wnt/β-catenin pathway and inducing sorafenib resistance (84). AR could suppress the formation of HCC vasculogenic mimicry (VM) by downregulating circRNA7/miRNA7-5p/VE-Cadherin/Notch4 signaling pathways in HCC, which will help in the design of novel therapies against HCC (100). In another study, under hypoxic conditions, AR can suppress HCC invasion/metastasis by targeting CIRC-LNPEP/miR-532e3p/RAB9A signal axis (101). Estrogen receptor α (ERα) can suppress HCC cell invasion *via* altering the ERα/circRNA-SMG1.72/miR-141-3p/GSN signaling, and targeting this newly identified signaling with small molecules may help in the development of novel therapies to better suppress the HCC progression (102). Except this, there are certain small molecular activators or inhibitors targeting the circRNAs signaling pathways in the treatment of HCC, including HNF4a, RBM3, KIAA1429, DHX9, Twist1, nudix hydrolase 21 (NUDT21), andQKI5 (33, 38, 60, 61, 71, 75, 103). However, the technique of specifically targeting a specified circRNA in HCC patients still needs further study, and development of novel targeted therapies remains the priority in hepatocellular carcinoma (HCC) treatments.

High stability and abundant circRNAs in tissues and various body fluids make it able to serve as biomarker for early diagnosis and prognosis prediction of HCC patients. At the same time, exosomes can carry circRNAs from tumor cells to recipient cells mediate cell–cell communication to regulate the behavior of recipient cells, suggesting that circRNAs can be used as non-invasive circulating biomarkers for cancer diagnosis. However, only a small number of functional circRNAs have been identified in HCC, and most of these studies have focused on miRNA sponge or ceRAN mechanism. Therefore, identifying functional circRNAs; clarifying their biogenesis, cell location, and function; further understanding the relationship between circRNA and the etiology, development, and molecular mechanism of HCC; screening target genes and corresponding signal pathways will help to improve HCC diagnosis and prognosis prediction and provide practical and reliable basis for clinical therapy.

## Author Contributions

HZY wrote the manuscript. XY and CYF revised and approved the manuscript. All authors contributed to the article and approved the submitted version.

## Funding

This study was funded by Hong Kong Scholars Program (XJ2020012), National Natural Science Foundation of China (81902431), Excellent Youth Project of Natural Science Foundation of Heilongjiang (YQ2019H007), Special Project of China Postdoctoral Science Foundation (2019T120279), Special Project of Heilongjiang Postdoctoral Science Foundation (LBH-TZ1016), China Postdoctoral Science Foundation (2018M641849, 2018M640311), Heilongjiang Postdoctoral Science Foundation (LBH-Z18107 and LBH-Z18112), the Fundamental Research Funds for the Heilongjiang Provincial Universities (2018-KYYWF-0511, 2018-KYYWF-0498), Postgraduate Innovative Research Project of Harbin Medical University (YJSCX2016-21HYD), Foundation of Key Laboratory of Myocardial Ischemia, Ministry of Education (KF201810), and Chen Xiaoping Foundation for the Development of Science and Technology of Hubei Province (CXPJJH11800004-001, CXPJJH11800004-003).

## Conflict of Interest

The authors declare that the research was conducted in the absence of any commercial or financial relationships that could be construed as a potential conflict of interest.

## Publisher’s Note

All claims expressed in this article are solely those of the authors and do not necessarily represent those of their affiliated organizations, or those of the publisher, the editors and the reviewers. Any product that may be evaluated in this article, or claim that may be made by its manufacturer, is not guaranteed or endorsed by the publisher.
